# Synthesis and Characterization of High-Entropy Dawsonite-Type Structures

**DOI:** 10.1021/acs.inorgchem.3c00179

**Published:** 2023-03-13

**Authors:** Amy J. Knorpp, Pietro Allegri, Shangxiong Huangfu, Alexander Vogel, Michael Stuer

**Affiliations:** † Laboratory for High Performance Ceramics, Empa. Swiss Federal Laboratories for Materials Science and Technology, Überlandstrasse 129, CH - 8600 Dübendorf, Switzerland; ‡ Department of Applied Science and Technology, Politecnico di Torino, Corso Duca Degli Abruzzi 24, 10129 Torino, Italy; § Electron Microscopy Center, Empa, Swiss Federal Laboratories for Material Science and Technology, 8600 Dübendorf, Switzerland

## Abstract

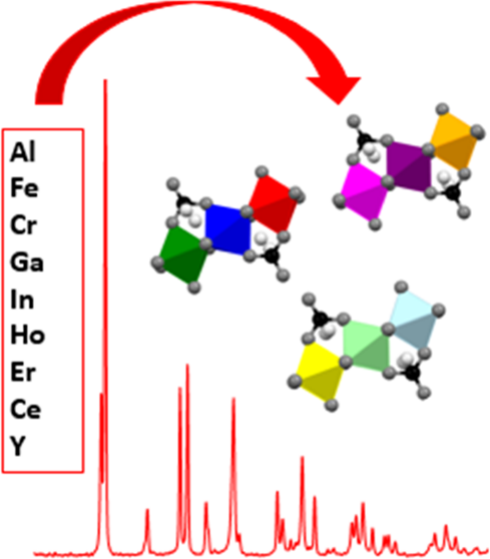

High-entropy hydroxides are an emerging subcategory of high-entropy materials (HEMs), not only because they can serve as tailorable precursors to high-entropy oxides (HEOs) but also because they can have unique high-entropy properties themselves. Many hydroxide crystal structures that are important for various applications are yet to be studied within the context of high-entropy materials, and it is unknown if they can take a high-entropy form (typically five or more incorporated cations). One such material is the dawsonite-type structure, which is a material with applications in both catalysis and ceramics. This work focuses on the adaptation of a dawsonite-type structure (NH_4_M(OH)_2_CO_3_) into a high-entropy material. Through a coprecipitation synthesis method, dawsonite-type materials readily took a high-entropy form with five cations that were equimolar and homogeneously distributed. The specific chemistries investigated were Al, Cr, Fe, and Ga with a fifth cation that was varied with increasing ionic radius (In, Er, Ho, Y, Eu, Ce, La). High-entropy dawsonites also exhibit the ″memory effects″ of non-high-entropy dawsonites. This work extends the field of high-entropy materials to include a structure that can serve as a material platform for the synthesis of high-entropy catalytic materials and ceramic powders.

## Abstract 2

A dawsonite-type structure readily adapts to a multicationic high-entropy form.

## 1 Introduction

High-entropy materials (HEMs) are an emerging field that has expanded well beyond its origins in metal alloys^1,2^ to now include oxides,^3^ carbides,^4^ nitrides,^5^ and silicides.^6^ The driving concept for high-entropy materials is similar across all material classes, where configurational entropy is typically increased during the synthesis by achieving equimolar concentration of elements, increasing the number of elements incorporated (typically more than five cations), and ensuring homogeneous distribution of elements in the structure. High-entropy oxides, a subcategory of high-entropy materials, are becoming an enticing material platform^7−9^ for a wide range of applications including catalysis,^10,11^ energy storage,^12,13^ and thermoelectric materials^14^ because of unexpected or improved properties from synergies between multiple cations (i.e., cocktail effects), severe lattice distortions, charge distortions, and even in some cases from entropy stabilization. Recently, high-entropy hydroxides like layered hydroxides (LDHs) have gained attention as high-entropy precursors to high-entropy oxides,^15−17^ as well as in use as high-entropy materials themselves for their magnetic,^18^ ionic conductivity,^19^ and electrocatalytic properties.^20^

Similar to LDHs, there are other structures that warrant investigation as potential high-entropy materials. One such material is the dawsonite-type structure, which has a wide range of industrial interest from fields ranging from ceramics^21−23^ to catalysis^24,25^ but which is yet to be investigated in a high-entropy form with five or more cations incorporated into the structure. Dawsonite (NaAl(OH)_2_CO_3_) is a naturally occurring mineral,^26^ and often its synthetic form is an ammonium analogue (NH_4_M(OH)_2_CO_3_),^27^ where M is often aluminum but can also be a variety of M(III) cations.^24,28,29^ The synthetic form of dawsonite with aluminum can also be referred to as aluminum ammonium carbonate hydroxide (AACH). Advantageous in the field of catalysis is the variety of cations that can be incorporated into its structure, thus allowing catalytically active sites to be dispersed and stabilized within a porous matrix.^29−32^ In the field of ceramics, dawsonite can be synthesized as a submicron powder with tailorable and uniform morphology, which can then be used as precursors for fine-grained ceramic powders,^24,27,33,34^ allowing for control of the microstructure.^23^

Given that high-entropy oxides and hydroxides are an emerging field for catalysis and ceramics, it is an opportune time to further identify and develop materials that can take high-entropy forms. This study demonstrates for the first time the adaptation of dawsonite-type materials into their high-entropy form with the incorporation of five cations. The high-entropy dawsonite-type structure shows immense flexibility for incorporating larger cations. High-entropy dawsonite-type structures with Al, Fe, Cr, Ga, In; Al, Fe, Cr, Ga, Er; Al, Fe, Cr, Ga, Ho; Al, Fe, Cr, Ga, Y; and Al, Fe, Cr, Ga, Ce were synthesized and characterized by scanning electron microscopy, energy-dispersive spectroscopy, X-ray powder diffraction, thermal analysis, nitrogen adsorption, and infrared spectroscopy.

## 2 Experimental Section

### 2.1 Multicationic Dawsonite-Type Synthesis

Synthesis of high-entropy dawsonite-type materials was done using a coprecipitation method similar to the one described by Li et al.^23,35^ and later adapted by Nam et al.^36^ for NH_4_AlCO_3_(OH)_2_. A metal nitrate solution was prepared with a total of 9 mmol of metal nitrates dissolved in 40 mL of deionized water. The total mmol of metal nitrates was kept constant, but to investigate whether dawsonite-type materials can take a high-entropy form, the metal nitrate solution included one, three, four, or five different M(III) nitrates. Specifically, synthesis was conducted for one cation (M = Al) as a reference, for three cations (M = Al, Fe, Cr), for four cations (M = Al, Fe, Cr, Ga), and for a combination of five cations where four cations were kept constant (M = Al, Fe, Cr, Ga) while the fifth cation was varied by increasing the ionic radius following the sequence In, Er, Ho, Y, Eu, Ce, and La. Depending on the number of cations, the mmol of each nitrate was adjusted to maintain equimolar equivalence between each metal cation. The exact quantities used for each formulation are shown in Table 1, and the ionic radii of the cations are also included.^37^

**Table 1:**
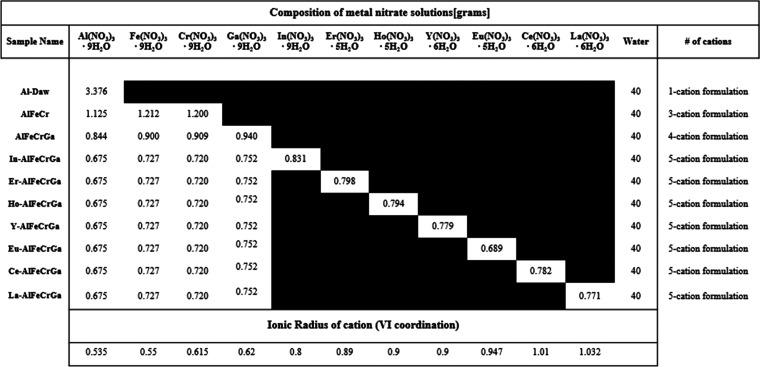
Composition of Metal Nitrate Solutions with One, Three, Four, and Five Different Cations^a^

a The ionic radii for the cations are included at the bottom of the table. Ionic radii are for VI coordination for the M^+3^ valence.

The supplier and purity of the nitrates salts were as follows: aluminum nitrate nonahydrate (Al(NO_3_)_3_·9H_2_O, Sigma-Aldrich, >98% purity), chromium nitrate nonahydrate (Cr(NO_3_)_3_·9H_2_O, Sigma-Aldrich, 99% purity), iron nitrate nonahydrate (Fe(NO_3_)_3_·9H_2_O, Sigma-Aldrich, >98% purity), gallium nitrate hydrate (Ga(NO_3_)_3_·*x*H_2_O, Sigma-Aldrich, 99.9%), indium nitrate hydrate (In(NO_3_)_3_·*x*H_2_O, Sigma-Aldrich, 99.9% purity), erbium nitrate pentahydrate (Er(NO_3_)_3_·5H_2_O, Sigma-Aldrich, 99.9% purity), holmium nitrate pentahydrate (Ho(NO_3_)_3_·5H_2_O, Sigma-Aldrich, 99.9% purity), yttrium nitrate hexahydrate (Y(NO_3_)_3_·6H_2_O, Sigma-Aldrich, 99.8% purity), europium nitrate pentahydrate (Eu(NO_3_)_3_·5H_2_O, Sigma-Aldrich, 99.9% purity), cerium nitrate hexahydrate (Ce(NO_3_)_3_·6H_2_O, Sigma-Aldrich, 99.99% purity), and lanthanum nitrate hexahydrate ((LaNO_3_)_3_·6H_2_O, Sigma-Aldrich, 99.9% purity).

As a separate solution, 0.06 mol of ammonium bicarbonate (>99% purity, Sigma-Aldrich) was mixed with 75 mL of deionized water. The solution was then adjusted to pH 9.5 with ammonium hydroxide solution (32% NH_3_ basis, Sigma-Aldrich), which required approximately 3.5 mL of NH_4_OH solution. Finally, the metal nitrate solution was added dropwise (6 mL/min) to the ammonium bicarbonate/ammonium hydroxide solution. The combined solution was stirred overnight (approximately 20 h) at room temperature. The resulting slurry was centrifuged, and the precipitant was collected and dried overnight at 333 K.

To test whether high-entropy dawsonite-type materials have similar memory effects to synthetic aluminum-dawsonite, a procedure similar to that described by Stoica et al. was used.^38^ The high-entropy dawsonite-type sample (In-AlFeCrGa) was calcined at 523 K overnight and approximately 0.3 g of the calcined material was stirred overnight at 323 K in 50 mL of 1 M (NH_4_)_2_CO_3_ (>99%, Sigma-Aldrich).

### 2.2 Characterization Methods

X-ray powder diffraction patterns were acquired on a PANalytical X’Pert PRO diffractometer with Cu Kα radiation with Ni filter. The Rietveld refinement analysis^39^ of the diffraction patterns of all high-entropy samples was performed with the package FULLPROF SUITE^40,41^ (version March-2019). The reflections of a main phase were indexed with an orthorhombic cell in the space group **Cmcm** (No. 64). The structural model was taken from the single-crystal X-ray diffraction refinement. Refined parameters were as follows: scale factor, zero shift, lattice parameters, metal atomic mass, and peak shapes as a Pseudo-Voigt* Axial divergence asymmetry function.

Scanning electron microscopy and elemental analysis were carried out on a Tescan SEM Vega3 fitted with a Bruker XFlash 6-10 detector with an accelerating voltage of 20 kV. For energy-dispersive X-ray (EDX) analysis, the powder was pressed into a pellet, affixed in epoxy, and polished to 1 μm using diamond lapping films. Bulk chemistry was taken of five different areas that were 200 μm by 100 μm in size. The average and standard deviation were then calculated. High-resolution EDX mapping was obtained on a Zeiss Gemini 460 with an Ultim max EDS detector and an accelerating voltage of 15 kV. An FEI Titan Themis operated at 300 kV was used for high-angle annular dark-field scanning transmission electron microscopy (HAADF-STEM) and energy-dispersive X-ray spectroscopy (EDS) mapping. For HAADF-STEM, a probe semiconvergence angle of 24 mrad was set in combination with an annular semidetection range of 66–200 mrad for the annular dark-field detector.

Thermogravimetric analysis (TGA) was performed on a Netzsch STA 449 f3 Jupiter instrument up to 600 °C with a heating rate of 10 °C/min under synthetic air flow. Fourier transform infrared (FTIR) spectra were collected at room temperature in the range of 320–4000 cm^–1^ with a Bruker Tensor 27 that was equipped with a diamond ATR. An average of 32 scans were taken with a resolution of 4 cm^–1^. Prior to nitrogen adsorption measurements, the samples were degassed under vacuum at 373 K overnight, and N_2_ isotherms were measured on a Microtrac Belsorp Mini X.

## 3 Results and Discussion

### 3.1 Structure Identification

**Figure 1:**
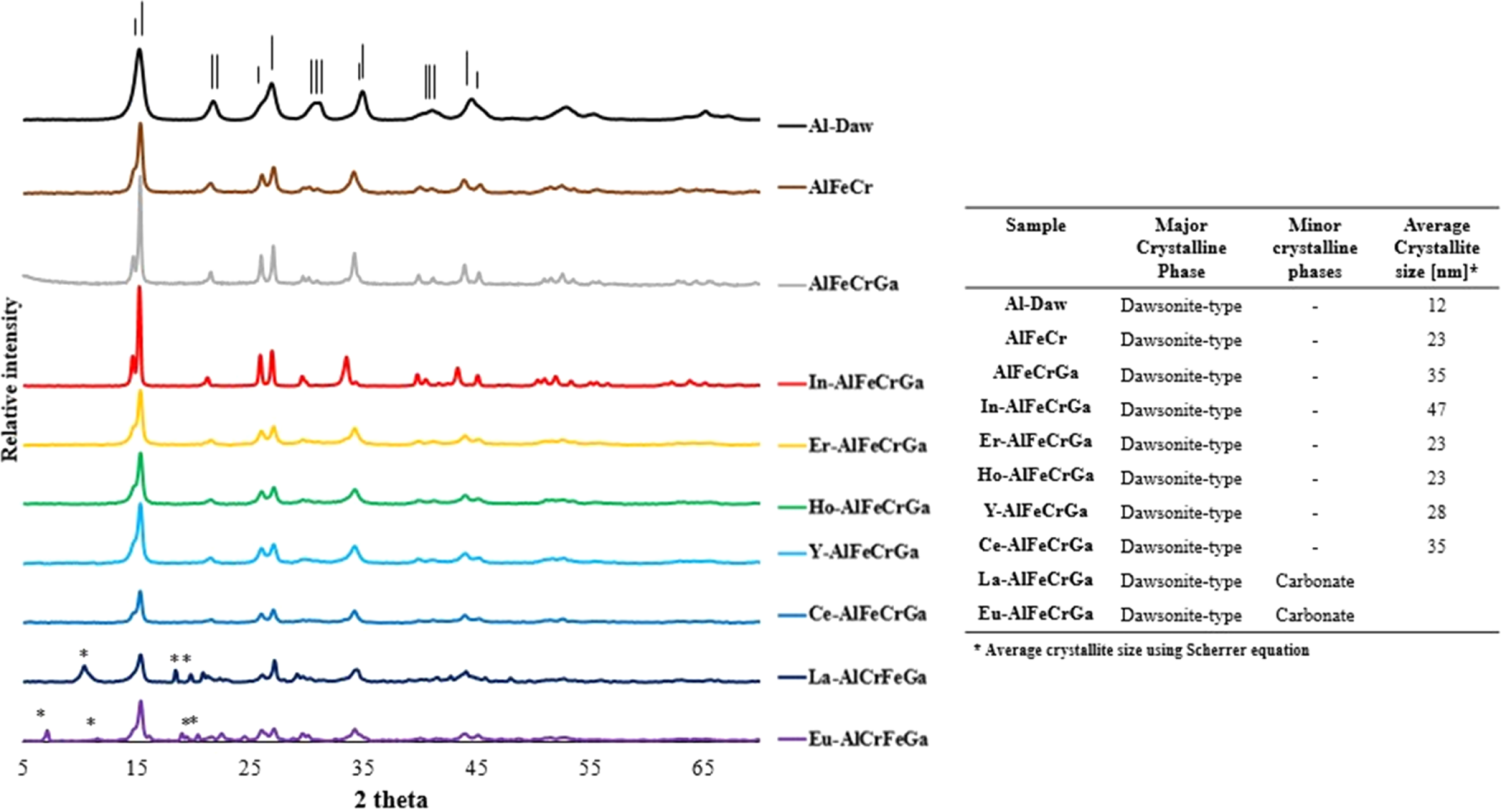
(Left) Powder X-ray diffraction patterns for dawsonite-type materials with one, three, four, and five different cations. Lines at the top represent reflection characteristics for dawsonite-type materials. * indicates additional phases. (Right) Summary of major and minor phases identified and calculated average crystallite sizes.

To verify the presence of a dawsonite-type structure, X-ray powder diffraction patterns were collected on the synthesized materials, as shown in Figure 1. All samples have characteristic reflections for an ammonium dawsonite-type structure (JCPDA 42-250), with the strongest three peaks being at 2θ = 15.4, 26.9, and 35.1°, which correspond to the (110), (200), and (221) reflections, respectively. No additional phases were observed for Al-Daw, AlFeCr, AlFeCrGa, In-AlFeCrGa, Er-AlFeCrGa, Ho-AlFeCrGa, Y-AlFeCrGa, and Ce-AlFeCrGa. Average crystallite sizes were calculated using the Scherrer equation^42^ based on the (110) reflection at approximately 15.4°. Between the high-entropy dawsonite-type samples, no clear trend is observed as the fifth cation is varied. The multicationic dawsonite-type structures appear to have an increase in crystallite size compared to the single-cation Al-Daw sample. Similar increases in crystallite size have been observed in studies with two cations (yttrium and aluminum),^43^ and this trend was attributed to yttrium ions slowing down the dawsonite crystallite growth. For two of the largest cations in this study, lanthanum and europium, reflections from additional phases were observed, specifically carbonate crystalline phases at 2θ = 10.3 and 18.3°. The resulting crystallographic parameters for the high-entropy dawsonite-type structure are presented in Table 2.

**Table 2:** Calculated Crystallographic Parameters for Multicationic Dawsonite-Type Structure

**crystallographic parameters**							
**sample name**	***a* (Å)**	***b* (Å)**	***c* (Å)**	**volume (Å**^**3**^**)**	***R***_**p**_ **(%)**	***R***_**wp**_ **(%)**	**χ**^**2**^
In-AlFeCrGa	6.6044(3)	12.048(1)	6.0147(3)	478.61(3)	5.56	8.19	7.30
Er-AlFeCrGa	6.5779(7)	12.025(2)	5.8681(5)	464.14(9)	2.42	3.14	1.31
Ho-AlFeCrGa	6.5757(9)	12.021(2)	5.8697(6)	464.0(1)	2.39	3.13	1.27
Y-AlFeCrGa	6.576 8(9)	12.014(2)	5.8674(6)	463.6(1)	2.33	3.05	1.30
Ce-AlFeCrGa	6.5 838(3)	12.025(2)	5.8699(4)	464.75(6)	2.74	3.55	1.20

**Figure 2:**
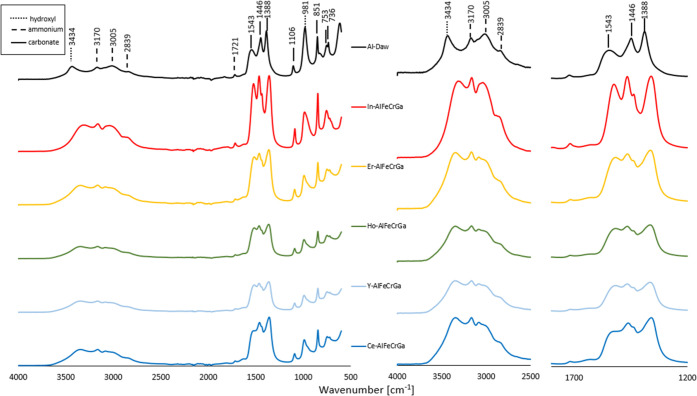
Fourier transform infrared (FTIR) spectra of synthesized multicationic dawsonite-type materials. (Left) Overview from 600 to 4000 cm^–1^. (Middle and right) Magnified view of regions of the FTIR spectra.

Fourier transform infrared spectra were collected for samples In-AlFeCrGa, Er-AlFeCrGa, Ho-AlFeCrGa, Y-AlFeCrGa, and Ce-AlFeCrGa as well as Al-Daw for reference, and the spectra are shown in Figure 2. Absorption was observed due to the bond vibrations of hydroxyl, ammonium, and carbonate groups and is consistent with a dawsonite-type structure.^44^ When comparing the single-cation Al-Daw to the multicationic dawsonite-type materials, significant shifts were observed for vibrations from hydroxyl and carbonate groups. Specifically, the *v*OH at 3434 cm^–1^ is red-shifted to ∼ 3345 cm^–1^ for all multicationic materials, which could indicate the strengthening of the hydrogen bond interaction in the multicationic dawsonite-type materials. Similar shifts have been observed in studies on Cr-dawsonite-type structures.^24^ Absorption events due to carbonate species (i.e., *v*_3_ C–O at 1543 and 1388 cm^–1^) are also shifted to the right, and the *v*_3_ C–O at 1446 cm^–1^ appears as two absorption bands. With the incorporation of multiple cations, shifts could be expected as a result of both the many-interaction situation arising from the cation mix and lattice distortions that result from the integration of cations of varying sizes.

### 3.2 High-Entropy Configuration

**Figure 3:**
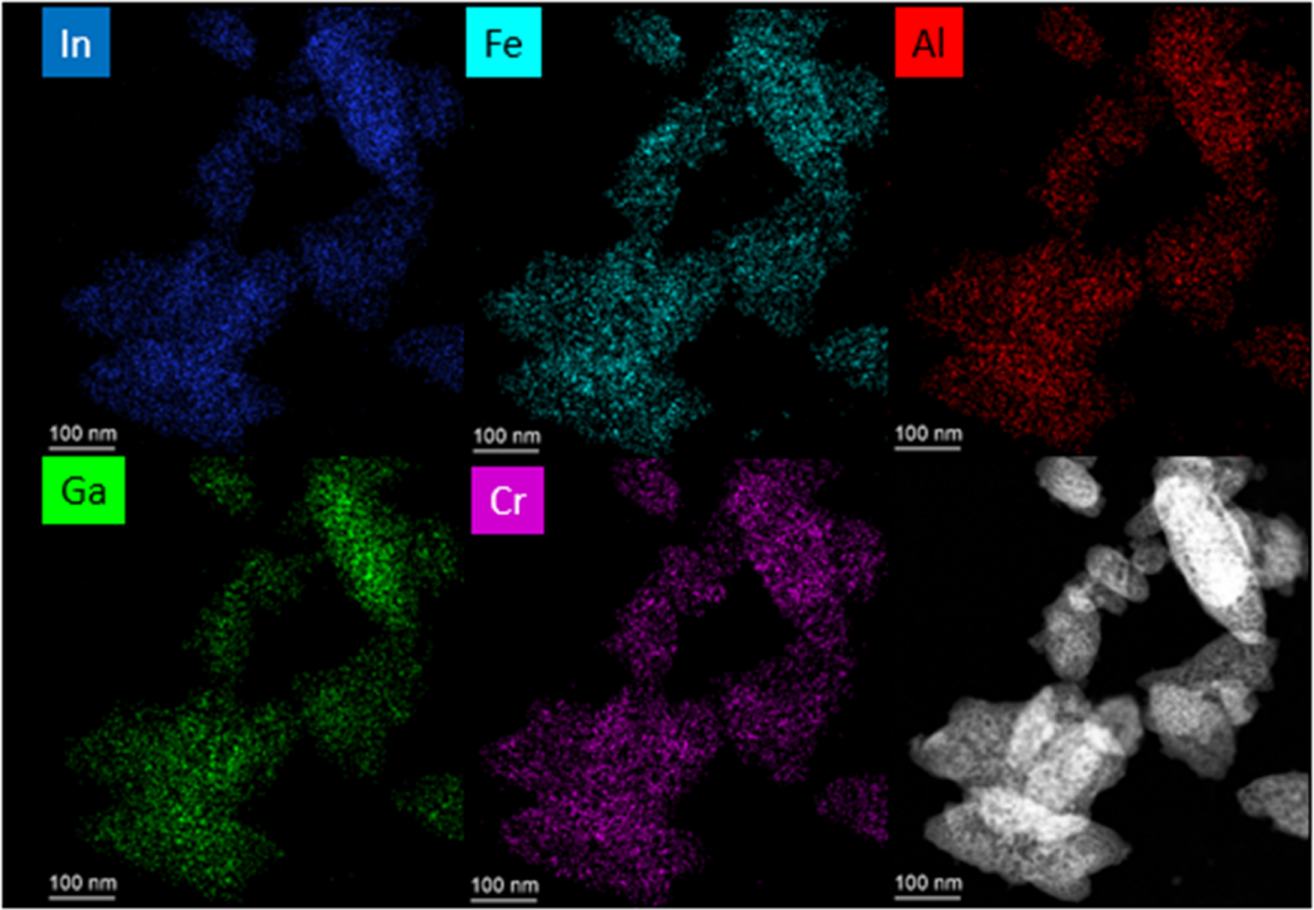
STEM image and EDS mapping for the In-AlFeCrGa sample.

To maximize configuration entropy, materials should have a homogeneous distribution of elements and a near-molar equivalent concentration of cations. Figure 3 shows a scanning transmission electron microscopy (STEM) image with energy-dispersive X-ray spectrometry (EDS) mapping for the In-AlFeCrGa sample. At this high resolution, the five cations (InAlCrFeGa) appear to be well distributed throughout the particle. However, this multicationic dawsonite-type material appears to have sensitive damage from the electron beam, and examples of this damage are shown in the Supporting Information in Figure S1.

**Figure 4:**
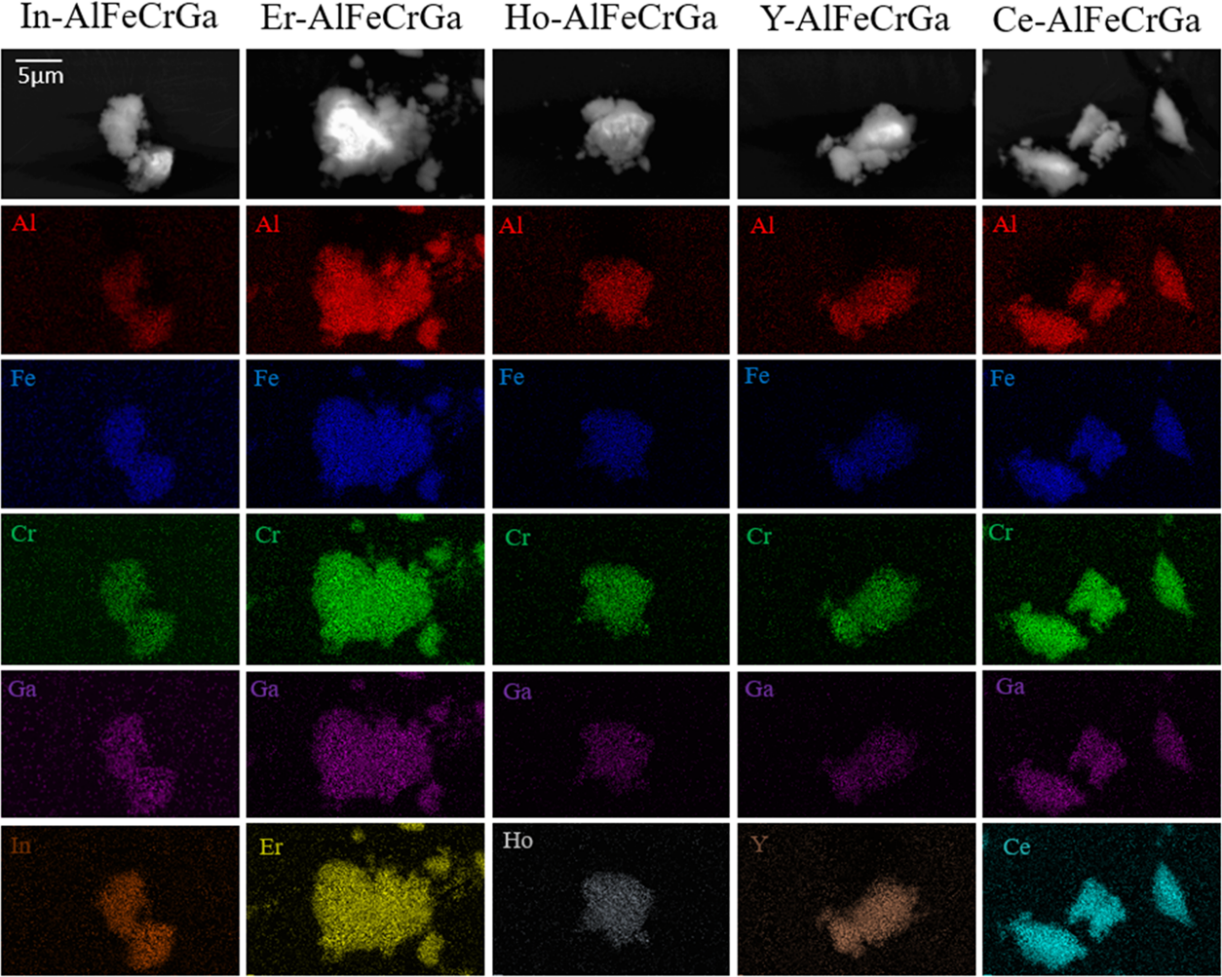
Elemental mapping of multicationic dawsonite-type material.

Additionally, elemental maps collected by SEM with EDS are shown in Figure 4. In-AlFeCrGa, Er-AlFeCrGa, Ho-AlFeCrGa, Y-AlFeCrGa, and Ce-AlFeCrGa appear to have a uniform distribution of cations; no element segregation was observed. Element analysis was also conducted by EDX analysis, and the atomic concentration of cations are presented in Table 3, all of which appear to have a near equimolar concentration for the five cations.

**Table 3:**
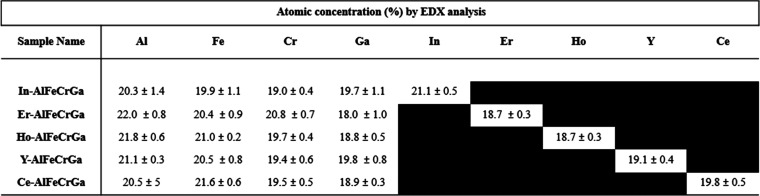
Atomic Concentration of Cations in Multicationic Dawsonite-Type Materials^a^

a The five measurements (200 μm by 100 μm in size) were analyzed for each pressed pellet of powder.

The dawsonite-type structure readily forms with five cations for a wide range of ionic radii as the fifth cation. The synthesized samples In-AlFeCrGa, Er-AlFeCrGa, Ho-AlFeCrGa, Y-AlFeCrGa, and Ce-AlFeCrGa satisfy the basic criteria for high-entropy materials. Depending on the definitions, however, these materials may not fall into subcategories such as entropy-stabilized, as they are not stable at high temperatures, but this is not to be expected due to their hydrated nature.

Additionally, new chemistries were accessed with the high-entropy dawsonite-type structures. Specifically, cations such as elements in the lanthanide group (i.e., Ho, Ce, and Er) show pure dawsonite-type structures when included in the synthesis. Previously, it had been shown to be difficult to incorporate lanthanide elements like holmium into dawsonite-type structures;^28^ because of possible mutually exclusive pH regimes for the formation of MO(OH)_2_^–^ and HCO_3_^–^, both ions are needed for the formation of dawsonite (eq 1), where hydrated carbonate or carbonate hydroxides form instead.
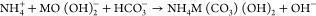
1In this study, only europium and lanthanum formed additional carbonate co-phases of dawsonite. It should be noted that pure dawsonite-type material formed with cerium despite it having radii and electropositivity between those of europium and lanthanum. However, lanthanum has been shown previously to incorporate into a two-cation (Al-La) dawsonite-type structure^25^ under slightly different synthesis conditions than this study. Specifically, there was an initial acidification step for the metal nitrates, and the synthesis was conducted at lower pHs than in this study. Therefore, it can be hypothesized that a high-entropy dawsonite-type structure is even more versatile in the incorporation of cation than in this study, and under optimized synthesis conditions for specific elements of interest, a wide range of chemistry can be obtained in the high-entropy form.

### 3.3 Additional Characterization of High-Entropy Dawsonite-Type Materials

**Figure 5:**
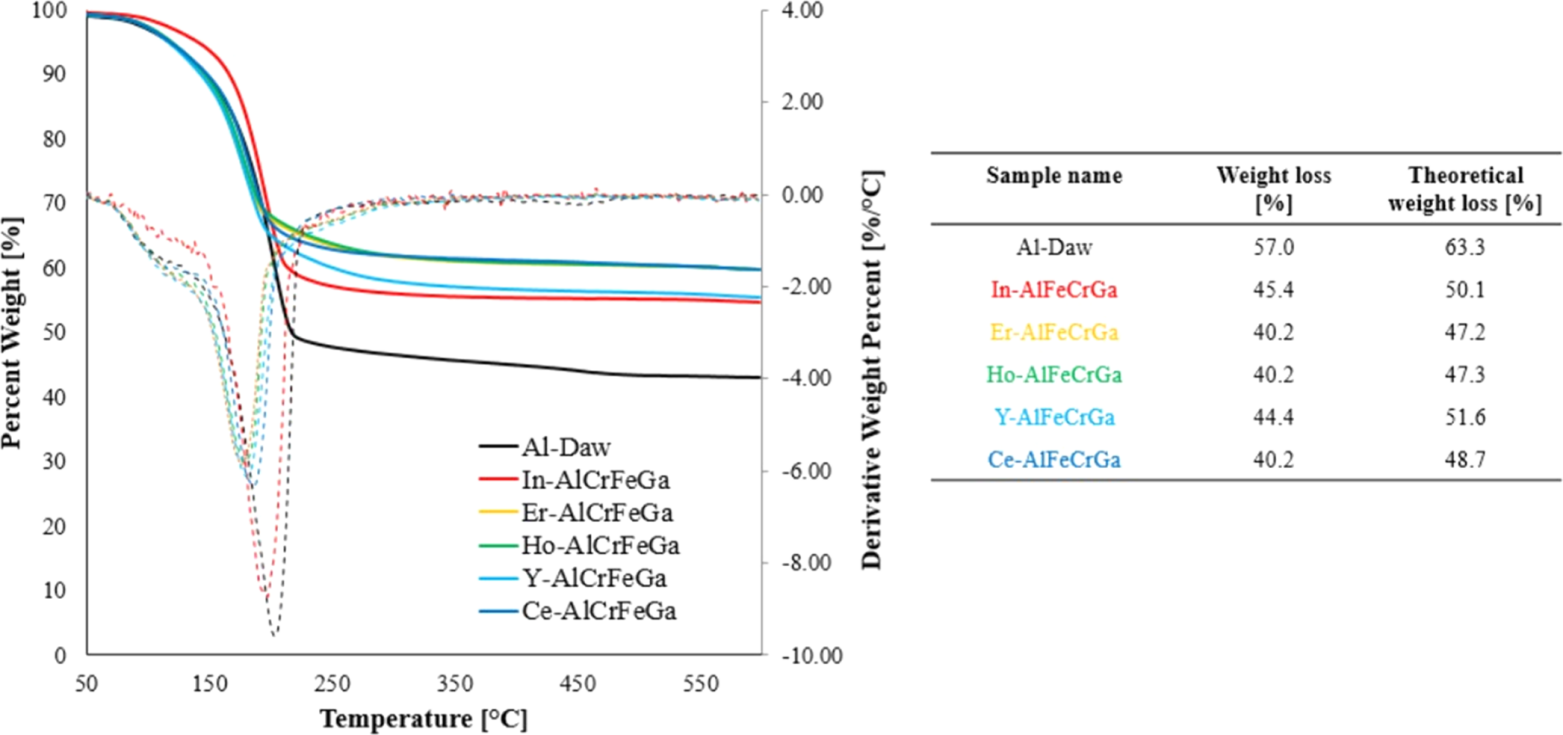
(Left) TGA profiles for high-entropy dawsonite-type materials. (Right) Weight loss as determined by TGA and theoretical weight loss for pure dawsonite-type material. Assumed calcined chemistry is M_2_O_3_, where M is the equimolar of the incorporated cations.

To further compare high-entropy dawsonite-type materials with their non-high-entropy forms like Al-Daw, additional characterization was carried out. In the literature, it is reported that synthetic dawsonite-type materials can also have amorphous material accompanying the crystalline phase.^45^ To account for amorphous phases, thermal analysis was conducted on the five high-entropy dawsonite-type samples. Figure 5 shows the weight loss between 50 and 600 °C, and theoretical weight loss for each sample was calculated based on the equimolar concentration of cations and an assumed M_2_O_3_ oxide structure after calcination. All samples had a 4–8% difference between the observed and calculated weight loss, which indicates that there are only small amounts of amorphous hydroxycarbonates, and the majority of elements are likely incorporated into the dawsonite-type structure. Since this amorphous fraction was not observed by elemental mapping, it likely also has a multicationic chemistry. The amount of amorphous fraction in this study is comparable to studies on the synthesis of non-high-entropy forms of dawsonite-type materials.^38,46^

**Figure 6:**
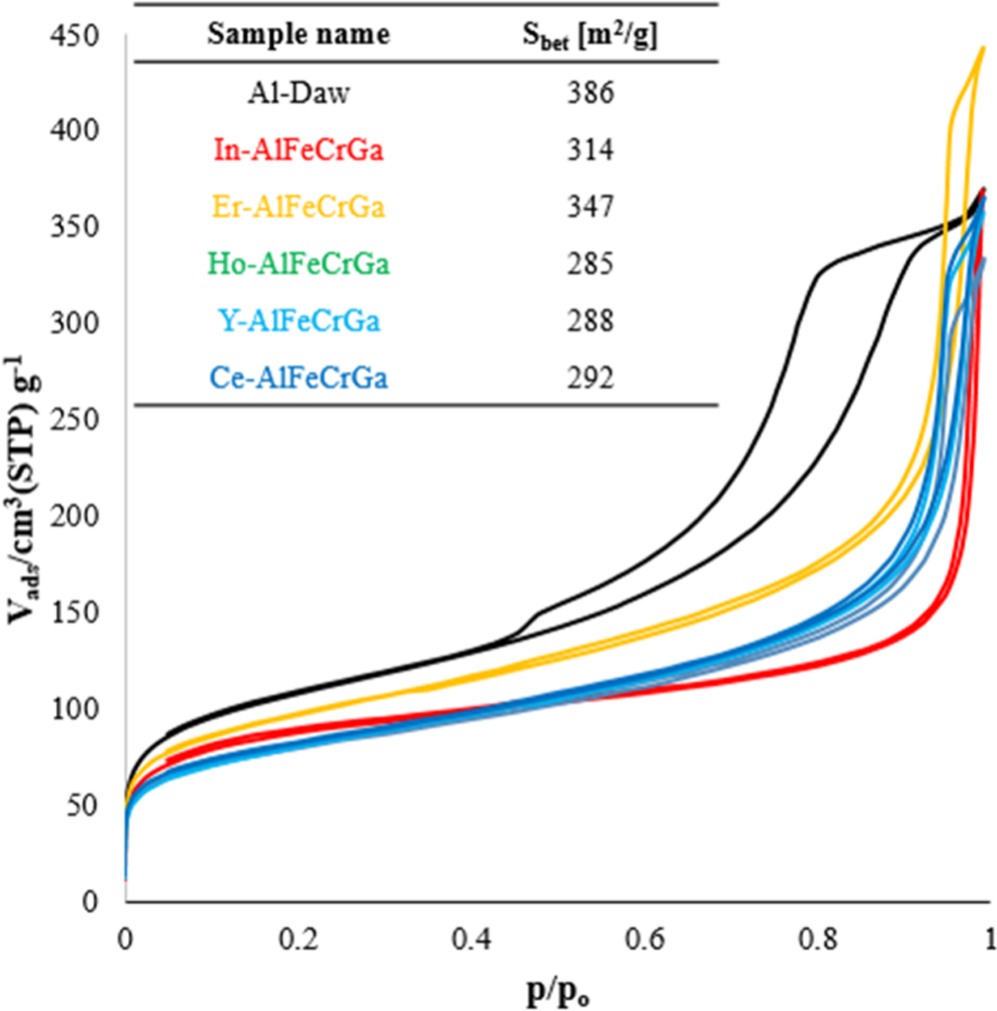
N_2_ adsorption and desorption isotherms. Brunauer–Emmett–Teller (BET) surface area values are shown in the inset table.

Nitrogen gas adsorption at 77 K for high-entropy dawsonite-type materials is shown in Figure 6. The Al-Daw isotherm resembles a type IV isotherm with an H1 hysteresis; however, the high-entropy samples do not exhibit this hysteresis but instead resemble a type II isotherm. Dawsonite-type materials have a wide range of surface areas reported in the literature, ranging from 100 to 800 m^2^/g, which can heavily depend on the synthesis method.^24,31,33^ In the case of high-entropy dawsonite-type materials, the surface area shows slightly lower values than the Al-Daw reference but generally falls within the expected range for the synthesis method used.

### 3.4 Memory Effect

The memory effect is well-documented for non-high-entropy forms of dawsonite-type materials.^38^ Specifically, it has been observed that calcination at 523 K results in an amorphous phase, but that dawsonite structure can be reconstructed completely by treating with an ammonium carbonate solution.

**Figure 7:**
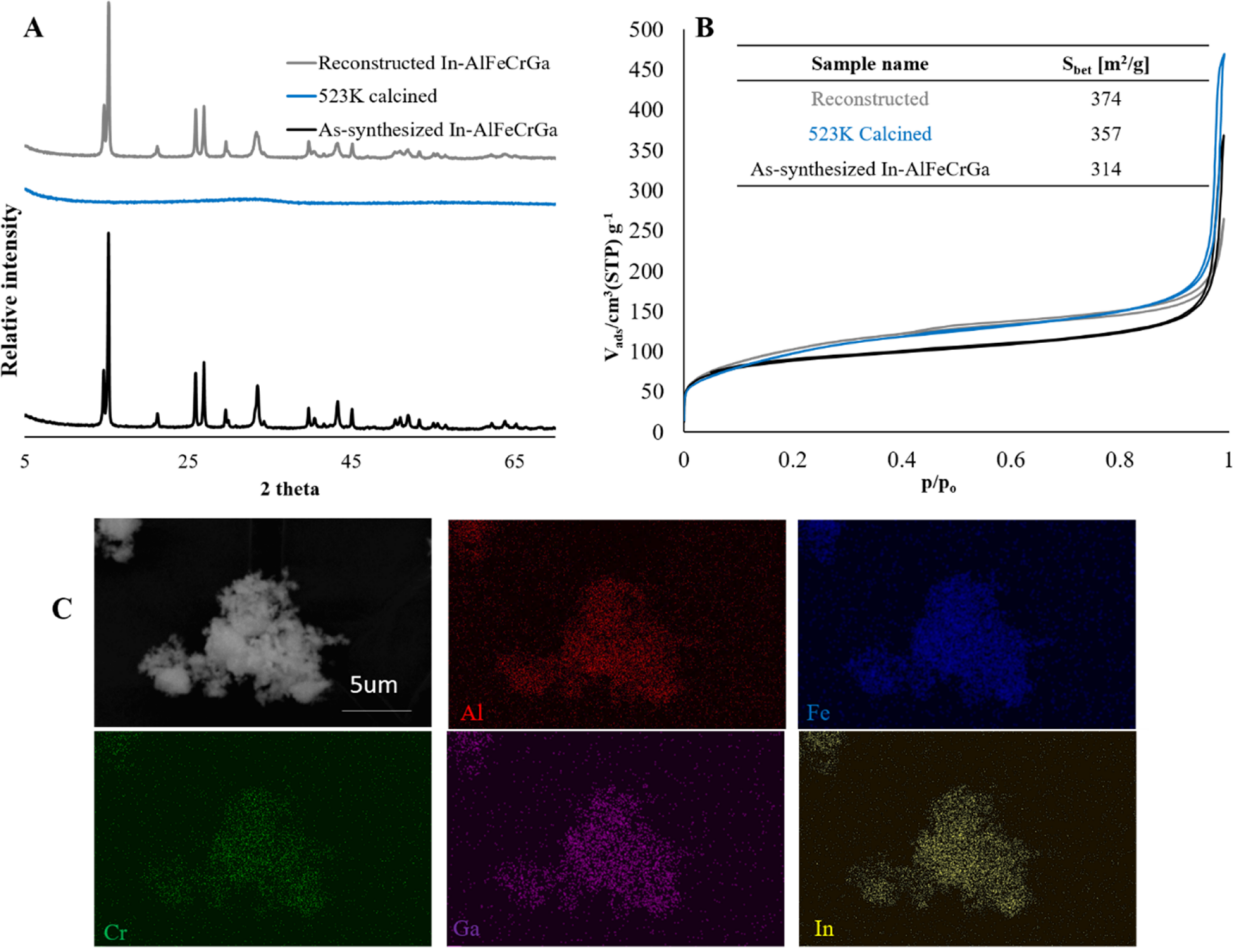
(A) X-ray powder diffraction of high-entropy dawsonite-type material as-synthesized, calcined at 523 K, and reconstructed by ammonium carbonate treatment. (B) Nitrogen adsorption and desorption on as-synthesized, calcined, and reconstructed high-entropy dawsonite-type materials (In-AlFeCrGa). (C) Elemental mapping by SEM/EDS for reconstructed high-entropy dawsonite-type materials with In, Al, Fe, Cr, and Ga.

The high-entropy dawsonite-type structure In-AlFeCrGa also exhibited this memory effect behavior with full reconstruction when treated with ammonium carbonate. Figure 7 shows an X-ray powder diffraction of In-AlFeCrGa as-synthesized, calcined at 523 K, and reconstructed. After calcination at 523 K, the characteristic diffraction pattern for dawsonite disappeared. After treatment with ammonium carbonate, the crystallinity again completely recovered. Similarly, high specific surface areas could also be maintained through the calcination and reconstruction of the high-entropy dawsonite-type material, with the specific surface area even slightly increasing from 314 to 373 m^2^/g when the material went through reconstruction (Figure 7). This increased porosity has also been observed for a non-high-entropy form of dawsonite.^38^ The reconstruction of the dawsonite-type structure also did not affect the homogeneous distribution of cations, and a similar distribution was observed by elemental mapping before (Figure 4) and after (Figure 7) ammonium bicarbonate treatment.

## 4 Conclusions

A dawsonite-type structure was synthesized and readily incorporated five cations with equimolar and homogeneous distribution, thus meeting the basic criterion for high-entropy materials and representing the first instance of this type of high-entropy material reported in the literature known to the authors. The chemistry of high-entropy dawsonite-type materials can be tailored by selecting a wide range of cations and cation sizes. In this study, high-entropy dawsonite-type materials were synthesized with a base of Al, Fe, Cr, and Ga, complemented with either In, Er, Ho, Y, or Ce as the fifth cation, accessing chemistries previously not available for dawsonite-type structures. Many common features for dawsonite-type materials could also be observed for their high-entropy form; in particular, they maintained a BET surface area of >200 m^2^/g and the ability to be reconstructed through a memory effect. The expansion of high-entropy materials to include high-entropy dawsonite-type structures represents an important step in the development of high-entropy materials targeted for specific catalytic applications and for ceramic powder synthesis, thanks to these materials’ porosity and tailorable surface and bulk chemistry.

## Supporting Information Available

HAADF-STEM image of beam damage in the electron beam on sample InAlCrFeGa and elemental maps from high-resolution SEM-EDS of the high-entropy dawsonite-type materials (PDF)
