# Supplementary material for: Synthesis and Characterization of High-Entropy Dawsonite-Type Structures
Source: Inorg Chem. 2023 Mar 13;62(12):4999–5007. doi: 10.1021/acs.inorgchem.3c00179 (PMC10052357; doi:10.1021/acs.inorgchem.3c00179)
Supplement: Supplementary file 1 — ic3c00179_si_001.pdf [file ic3c00179_si_001.pdf]

Supporting information:

Synthesis and characterization of high-entropy dawsonite-type structures

Amy J. Knorpp[1], Pietro Allegri[1,2], Shangxiong Huangfu[1], Alexander Vogel[3], Michael Stuer[1*]*

[1]Laboratory for High Performance Ceramics , Empa. Swiss Federal Laboratories for Materials Science and Technology Überlandstrasse 129 CH - 8600 Dübendorf, Switzerland. E-mail: amy.knorpp@empa.ch, michael.stuer@empa.ch

[2]Department of Applied Science and Technology, Politecnico di Torino, Corso Duca Degli Abruzzi 24, 10129, Torino, Italy

[3]Electron Microscopy Center, Empa, Swiss Federal Laboratories for Material Science and Technology, 8600 Dübendorf, Switzerland

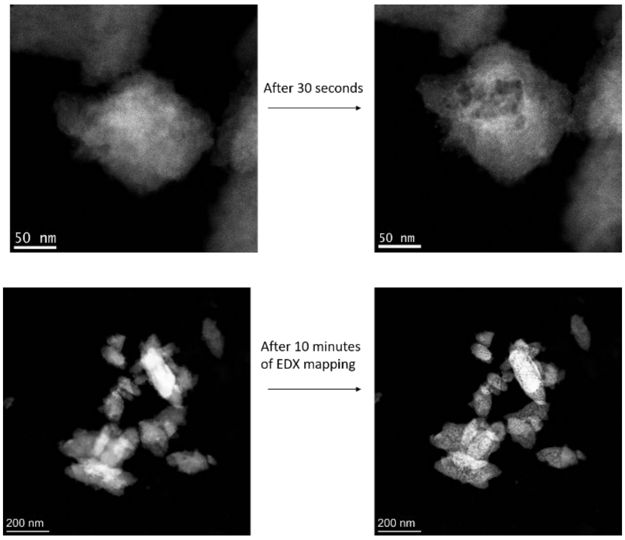

Figure S1: (top) HAADF-STEM image taken before and after 30second. (bottom) HAADF-STEM image before and after 10 minutes of EDX mapping. A beam current of 118 pA was used to reduce the electron-beam induced damage.

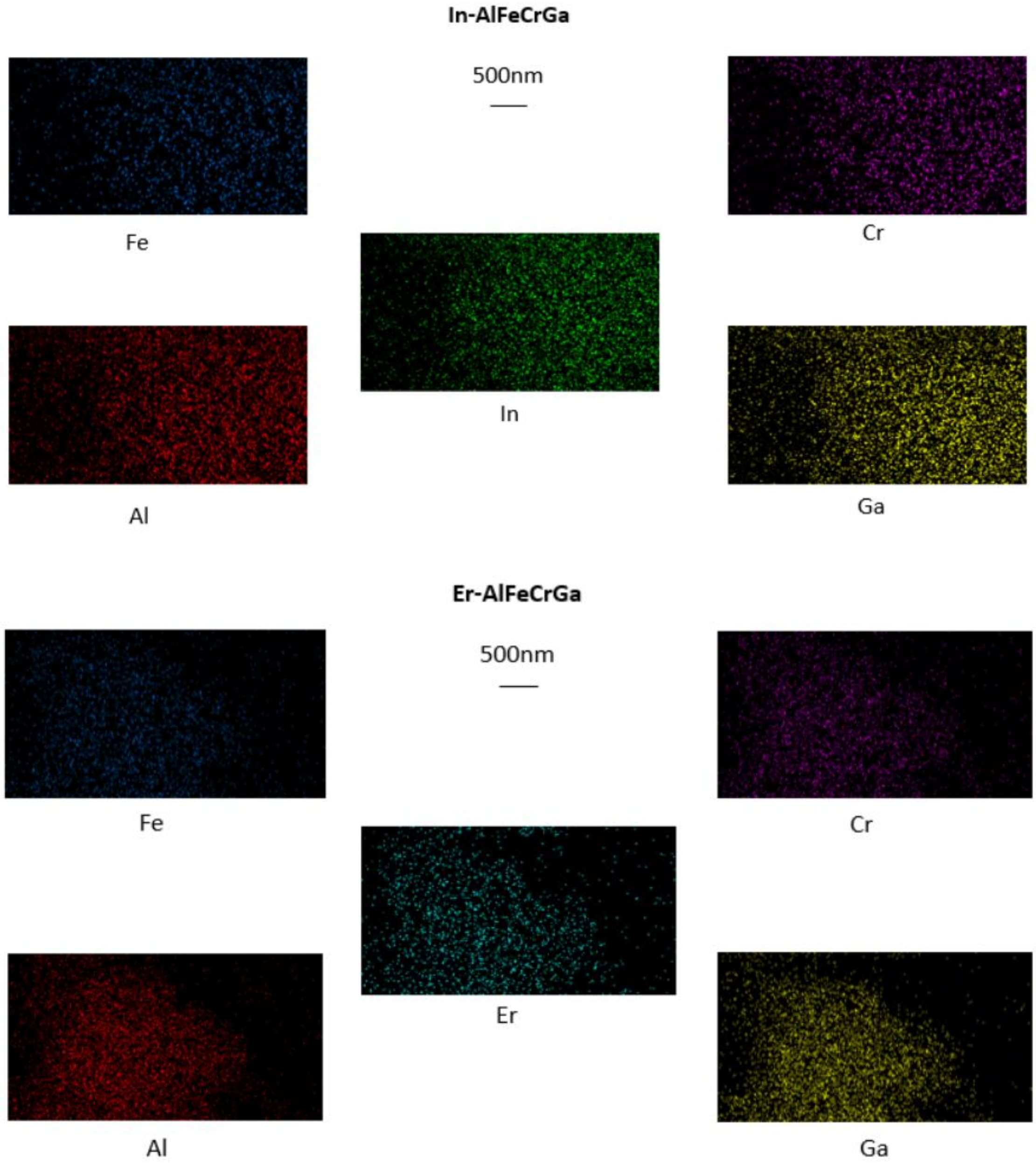

Figure S2: Elemental mapping on high-entropy dawsonite-type structures by SEM-EDS.

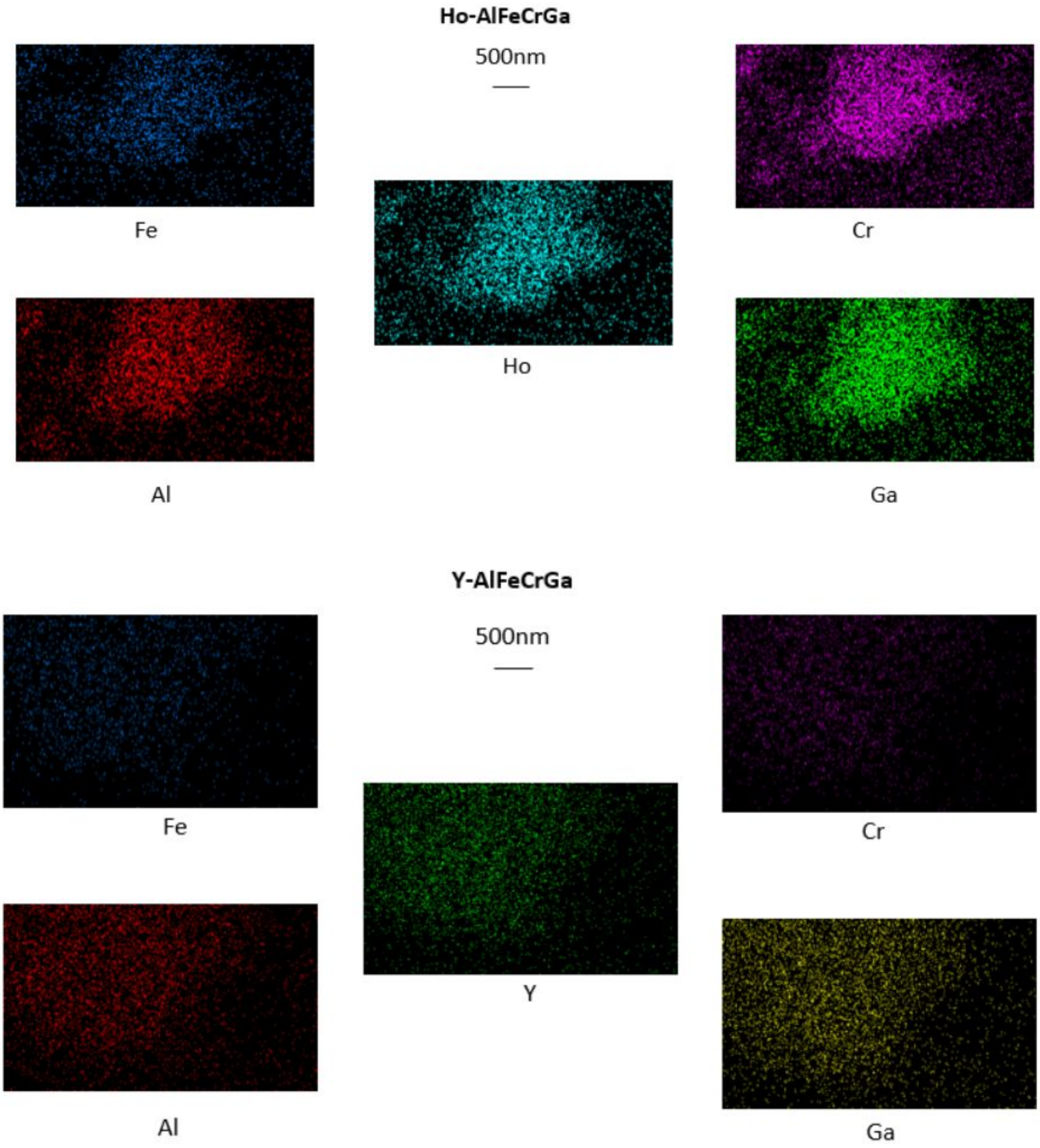

Figure S3: Elemental mapping on high-entropy dawsonite-type structures by SEM-EDS.

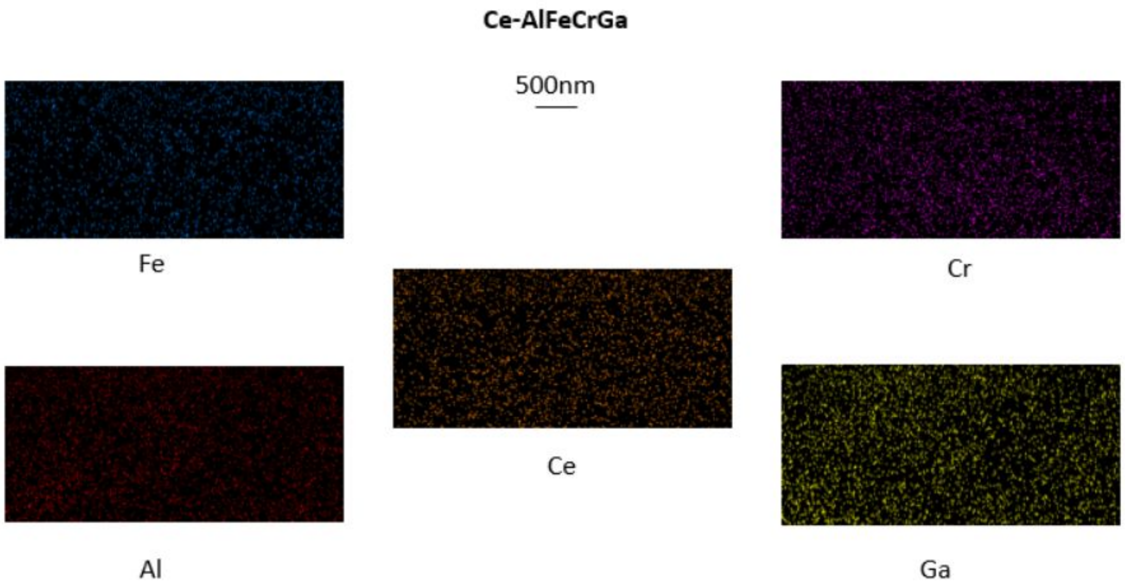

Figure S4: Elemental mapping on high-entropy dawsonite-type structures by SEM-EDS.
